# Supplementary material for: A Multimodal Spatial and Epigenomic Atlas of Human Adult Lung Topography
Source: bioRxiv. 2025 May 23:2025.05.23.655666. Preprint. [Version 1] doi: 10.1101/2025.05.23.655666 (PMC12140004; doi:10.1101/2025.05.23.655666)
Supplement: Supplement 3 [file media-3.zip › Supplementary Tables snHLA/Supplementary Data snHLA.pdf]

Supplementary Data

A Multimodal Spatial and Epigenomic Atlas of Human Adult Lung Topography

Thu Elizabeth Duong*, Dinh Diep, Kimberly Conklin, Indy Bui, Jeffrey M. Purkerson, Eric Boone, Jacqueline Olness, Sahil Patel, Beverly Peng, Colin Kern, Zoey Zhao, Ravi Misra, Heidie Huyck, Jamie Verheyden, Zea Borok, Yun Zhang, Richard Scheuermann, Quan Zhu[4], Gail Deutsch, James Hagood*, Xin Sun*, Kun Zhang*, Gloria S. Pryhuber*
*Corresponding Authors

Supplementary Table Legends

Supplementary Table 1. Donor Demographics and Clinical/Pathological Assessment.
Clinical metadata and histopathology for 11 healthy lung tissue donors used in this study.

Supplementary Table 2. Lung Block Anatomical Characterizations and Use in Assays.
Summary of omic experiments presented on 63 human lung donor blocks, including location, pathology review, and IDs for HuBMAP Portal data access.

Supplementary Table 3. snRNA cluster annotations and QC metadata.
Summary of cell type annotations, number of nuclei, mean UMI, and mean genes per lung block and donor for all snRNA clusters.

Supplementary Table 4. snHLA Cell Type and Marker Gene Dictionary.
Cell type annotations at class and different subclass levels with curated and NSForest marker genes for harmonized 10X snRNA and SNARE-RNA.

Supplementary Table 5. snHLA Cell Type Alignment with Cell Ontology IDs.

Supplementary Table 6. snRNA NS-Forest Marker Genes.
Summary of NS-Forest necessary and sufficient marker and binary genes at each annotation level.

Supplementary Table 7. Antibodies Used in Multiplexed Immunofluorescence.
Description of target protein antibodies used in MxIF panels including dilution factors.

Supplementary Table 8. SNARE2 RNA/AC cluster annotations and QC metadata.

Supplementary Table 9. Subclass level 3 cell type specific accessible regions and transcription factor motifs.
Differentially Accessible Regions (DARs), top ChromVAR transcription factor motifs, and top active (accessible and expressed) ChromVAR transcription factor motifs for subclass.L3 annotations.

Supplementary Table 10. MERFISH gene panel.
List of genes and codebook for MERFISH.

Supplementary Table 11. Summary of Cellchat receptor ligand interactions.

Supplementary Table 12. Subclass level 5 cell type specific accessible regions and transcription factor motifs.
Differentially Accessible Regions (DARs), top ChromVAR transcription factor motifs, and top active (accessible and expressed) ChromVAR transcription factor motifs for subclass.L5 annotations.
